# Takotsubo Cardiomyopathy and Autoimmune Disorders: A Systematic Scoping Review of Published Cases

**DOI:** 10.1155/2024/7259200

**Published:** 2024-02-20

**Authors:** Mohsen Farjoud Kouhanjani, Seyed Ali Hosseini, Seyedeh Maryam Mousavi, Zahra Noroozi, Paniz Sadeghi, Armita Jokar-Derisi, Mohammad Saleh Jamshidi Mouselou, Meysam Ahmadi, Armin Attar

**Affiliations:** ^1^Clinical Microbiology Research Center, Shiraz University of Medical Sciences, Shiraz, Iran; ^2^School of Medicine, Shiraz University of Medical Sciences, Shiraz, Iran; ^3^Student Research Committee, School of Medicine, Shiraz University of Medical Sciences, Shiraz, Iran; ^4^Department of Cardiology, School of Medicine, Namazi Teaching Hospital, Shiraz University of Medical Sciences, Shiraz, Iran

## Abstract

**Introduction:**

Takotsubo cardiomyopathy (TCM) features transient left ventricular apical dysfunction or ballooning. The underlying mechanism remains elusive; however, evidence suggests the role of different physical and psychological stressors. We systematically reviewed patients presenting with TCM and autoimmunity to explore the link between the two conditions.

**Methods:**

We applied the Preferred Reporting Items for Systematic reviews and Meta-Analyses extension for Scoping Reviews (PRISMA-ScR) to report this review. Using keywords related to autoimmune/immune-mediated diseases and TCM, we searched PubMed, Scopus, and WOS in March 2022. The final results were added to a data extraction sheet. Data were analyzed by SPSS version 26.0.

**Results:**

Our search yielded 121 studies, including 155 patients. Females were considerably predominant. Most patients had a history of autoimmune disease, and almost a third had a history of cardiovascular disease. Dyspnea and chest pain were the most common chief complaints. More than 70% of patients had experienced physical stress. Myasthenia gravis, systemic lupus erythematosus, and multiple sclerosis were the most frequently reported autoimmune diseases.

**Conclusion:**

There were similarities in age and sex compared to classic TCM. TCM should be considered as a differential diagnosis for ACS, especially in patients with a positive background of autoimmunity. A precise reporting system is required for further studies.

## 1. Introduction

Takotsubo cardiomyopathy (TCM) [1, 2], also known as broken heart syndrome, apical ballooning syndrome, or stress cardiomyopathy, is a reversible dilating abnormality in the left ventricle's apical area resulting in systolic dysfunction [1, 3]. TCM was first described in 1990 in Japan, taking its name from its similar echocardiographic appearance to the wide-based, narrow-necked pots used traditionally to trap octopuses [4]. Its prevalence is 1–2.5%, mostly affecting postmenopausal women [1, 3]. Although the exact mechanisms remain elusive, the pathophysiology of TCM appears to involve microvascular dysfunction and catecholamine-induced cardiotoxicity [2]. Physical or emotional stressors, estrogen deficiency, and genetic factors can predispose individuals to this condition [1, 3].

Chest pain and dyspnea are TCM's most common clinical manifestations, while other serious presentations such as cardiogenic shock and ventricular fibrillation may also occur [5]. This condition mimics myocardial infarction, except that no coronary artery occlusion exists [1]. Typical features include cardiac enzyme elevations, wall motion irregularities, and electrocardiographic abnormalities—mostly T wave inversion [2]. Symptoms usually resolve spontaneously, but aggressive and invasive measures are required if hemodynamic instability develops [6]. TCM cases have a desirable recovery and prognosis, and hospital mortality rates vary between 1 and 2% [2].

The literature describes the coincidence of TCM with other conditions such as metabolic and endocrine dysfunctions [7], neurological diseases [8], psychiatric illnesses [9], and immune-mediated conditions [10, 11]. However, a major gap in research is the lack of a well-designed systematic review exploring the link between TCM and autoimmunity. Hence, considering the probable role of inflammation and autoimmunity in TCM [11], we designed this study to systematically review the clinical and para-clinical aspects of published cases with coincident TCM and autoimmune diseases.

## 2. Methods

**Figure 1:**
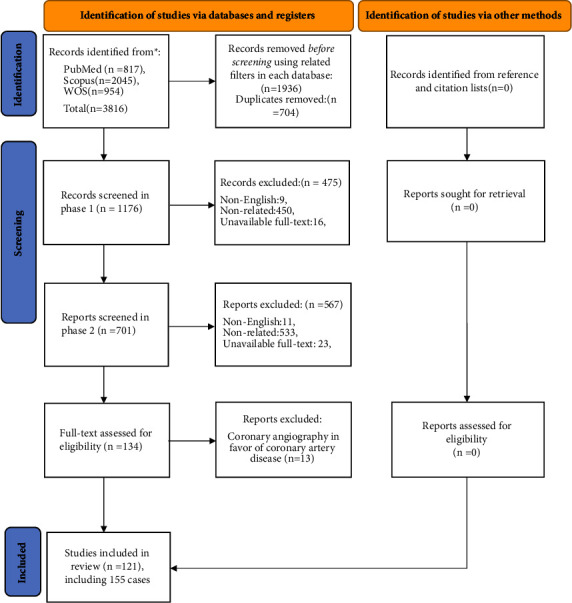
Systematic scoping review flowchart.

To report this systematic scoping review, we applied Preferred Reporting Items for Systematic reviews and Meta-Analyses extension for Scoping Reviews (PRISMA-ScR) [12]. We searched three major databases including PubMed, Scopus, and WOS in March 2022, using keywords related to autoimmune/immune-mediated diseases and TCM (Supplementary Material (available here)). Our search yielded 3816 articles. Using relevant filters on each database (*case-report* filter in PubMed and *article* filter for Scopus and WOS), we excluded 1936 articles. In the next step, the authors independently screened the results for eligible articles in two separate rounds (Figure 1).

Our inclusion criteria consisted of English articles of any type reporting TCM patients with autoimmune disorders. We excluded those with unavailable full texts and cases with abnormal findings in their coronary angiography in favor of coronary artery disease. We also removed duplicate cases, mainly reported in review articles.

After excluding duplications, the final results were added to a data extraction sheet. Data were analyzed by SPSS version 26.0. We used mean ± standard deviation (SD), median, and interquartile range (IQR) for continuous data, while frequency (percent) was used for categorical ones.

## 3. Results

Our review included a total of 155 patients with TCM associated with an autoimmune disease across 121 studies [13–133] (Supplementary Material (available here)), ranging in age from 4 to 84 years (Table 1). The median age was 60 (IQR: 42–71) years. Among females, the median age was 60, with an IQR of 43–70. Males had a slightly lower median age [58] with a wider IQR of 30–76 years. There was a strong female predominance, with a female-to-male ratio of around 5.3 to 1. Interestingly, men were younger by an average of 3.2 years. Although race was not reported in most cases, African-American, Caucasian, and White patients accounted for most of the reported races (Table 1).

**Figure 2:**
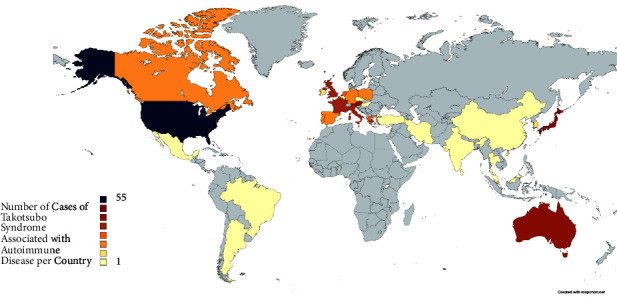
Global map of reported cases of Takotsubo cardiomyopathy associated with autoimmune disease. Created with https://mapchart.net.

In terms of continental distribution, most patients were from North America, followed by Europe and Asia. The distribution of cases by country is illustrated in Figure 1. Most (*n* = 55) cases were reported from the United States, after which came Japan (*n* = 12), Australia (*n* = 10), Austria (*n* = 10), the United Kingdom (*n* = 8), Italy (*n* = 8), and France (*n* = 8). The remaining countries reported four or fewer cases (Figure 2).

The past medical history of most (65.2%) patients was reported in the included studies (Table 2). When looking at the overall trends, most patients had a history of autoimmune disease, and almost a third of patients had a history of cardiovascular disease (any functional and/or structural heart disease, coronary artery disease, or hypertension). The autoimmune disease was often combined with cardiovascular disease (7.1%), other diseases (13.5%), or both (15.5%). While the proportion of patients with cardiovascular disease was almost identical (28%) in both genders, women had an almost three times higher rate of autoimmune disease (57.7% vs. 20.0%).

A summary of the clinical presentations of the patients included in this review is presented in Table 3. Almost a third of studies did not report a chief complaint, and roughly, a quarter of studies reported more than one chief complaint. The predominant chief complaint was dyspnea (46.9%) followed by chest pain (35.3%). Less common complaints (<10%) include syncope, palpitations, cyanosis, and cardiopulmonary arrest. Notably, the vital signs were not reported in roughly half the cases, with the other half having at least one abnormality. Tachycardia (23.6%) and hypotension (21%) were the most common abnormalities. An abnormal cardiovascular exam was reported in 21 cases; the remaining cases did not report any abnormalities.

In terms of the type of stress that triggered TCM, the type of trigger was unclear in 27 (17.4%) cases. Notably, 114 patients (73.5%) had a physical stress trigger, while 8 cases (5.2%) experienced psychological stress. Finally, 6 patients (3.9%) had both psychological and physical stress triggers.

Among the autoimmune diseases of patients with TCM, myasthenia gravis was the most common (18.8%), followed by systemic lupus erythematosus (14.2%), multiple sclerosis (11.6%), Guillain–Barre syndrome (9.7%), Grave's disease (7.8%), and rheumatic arthritis (5.2%) (Table 4). Autoimmune thyroiditis, autoimmune poly-glandular syndrome type II, vasculitis, and inflammatory bowel disease were also fairly common, each affecting 3.2% of cases (Table 4).

An autoimmune marker was not reported in 117 cases (75.5%). Among the 38 cases where it was reported, 6 cases (15.8%) were negative and 32 were positive (84.2%).

Abnormal laboratory data were not reported in 108 cases (69.7%). Elevated levels of ESR and CRP were reported in 6 and 12 cases, respectively. Increased BNP was reported in 16 cases. CKMB elevated in 25 cases. Troponin level was not measured/reported in 36 (23.2%) cases; among those whose troponin level had been checked, 7 cases (4.5%) were normal, while 111 patients (71.6%) had elevated levels, and one case had a borderline result.

The electrocardiogram (ECG) study was not reported for 27 (17.4%) cases. Isolated ST elevation, isolated ST depression, and isolated T inversion were reported in 22 (14.2%), 4 (2.6%), and 21 (13.5%) cases, respectively. The ECG was normal in 7 (4.5%) cases. Overall, T-inversion was the most common finding (39.2%), followed by ST elevation (34.2%) and nonspecific changes (34.8%). Interestingly, ST depression was noted in only 8.2% of cases.

Fifty-seven cases had no angiography report, and 98 patients had normal angiography findings. The echocardiography findings were not reported in 27.1% of cases (Table 5). Almost all reported cases (112 out of 113, 99.1%) had a reduced ejection fraction (EF) in the initial echocardiography study. The first EF varied between 10 and 71%, with the EF after recovery varying between 36 and 78%. Among those who had a recovery EF reported, 103 out of 110 (93.6%) achieved a normal EF. The median time to recovery was 18.50 (IQR: 10.00–42) days (Table 5.)

The final outcome of 9 patients was unclear, while 133 (85.8%) survived and 13 (8.4%) succumbed to their disease. The mean age of cases that resulted in fatalities was 64.31 ± 14.59 years, ranging from 31 to 81 years old. Eight patients (61.53%) were females and five (38.46%) were males. MG (in five cases) and SLE (in four cases) were the most frequently reported autoimmune disorders in this group. The presenting ejection fraction was reported in only six of these cases, with a mean value of 30.67 ± 12.37%, ranging from 12 to 47%.

## 4. Discussion

We systematically reviewed the clinical presentations, laboratory data, ECG, and echocardiographic findings of published cases of TCM associated with an autoimmune disease. Our results show that this association occurs predominantly in women in postmenopausal ages; this is in agreement with other studies indicating that the female sex is a strong risk factor for both disorders [134–136]. Conversely, a study in Japan showed TCM to be more prevalent among men for unclear reasons [137], while another study reported TCM occurring in men at younger ages [134].

Many studies did not report the race of the patients; however, Whites, Caucasians, and African-Americans comprised the majority of those with known ethnicity. We could not find any reports on the definite prevalence of TCM among different ethnicities. However, evidence suggests worse in‐hospital complications among African-Americans [138]. This parallels a study by Dias, Franco, Ross, and Hebert in terms of hospital length of stay. Interestingly, African-Americans' long-term prognosis has been better than Whites [139].

Almost a third of patients had a history of cardiovascular disease, and the proportion was the same in both genders. A systematic review of more than a thousand TCM patients reports a 54% prevalence of HTN [140]. In addition to HTN, other risk factors of cardiovascular diseases including diabetes, hyperlipidemia, obesity, and chronic kidney disease can affect TCM [141].

Myasthenia gravis (MG) was the most common autoimmune disease associated with TCM, followed by systemic lupus erythematosus (SLE), multiple sclerosis, Guillain–Barre syndrome (GBS), Grave's disease, and rheumatic arthritis (RA). Limited data are available on such co-occurrences. For instance, more than 15% of MG patients may develop cardiac involvements [142, 143]. Cardiac complications are also common in SLE [144]. In a review of the association of TCM and rheumatic disorders, 5 out of 16 were diagnosed with SLE [11]. Despite few published cases of concurrent TCM and MS, the presence of medulla oblongata lesions was a common finding, suggesting a potential role for catecholamines [145]. One review study supports TCM as a probable complication of GBS, especially for those with dysautonomia [146]. TCM may also present as a manifestation of thyrotoxicosis, especially Grave's disease [7, 147]. In one review study, RA was the second most frequent concurrent rheumatic disorder with TCM [11].

Emotional and physical stressors alone or in combination may trigger the development of TCM [148]. Although most previous studies have focused on the role of emotional stressors [149], physical stressors reined emotional ones in our study. Most of our patients presented with chest pain and dyspnea, agreeing with a recent review on TCM [1]. Tachycardia and hypotension were frequently observed; however, we could not find any study comprehensively addressing a detailed physical examination.

Most cases had elevated troponin levels, compatible with a review study on TCM [150]. BNP and CK-MB were positive in about 10% and 15% of cases, respectively. Unlike acute MI, BNP elevation is a universal finding in TCM [151, 152]. Studies are addressing the diagnostic value of NTproBNP/TnI, BNP/TnT, and BNP/CK-MB and ratios, especially in discriminating TCM from acute MI [153, 154].

Not only the clinical presentations but also the electrocardiographic findings may be misleading in differentiating TCM from acute coronary syndromes (ACSs) [155]. Keeping in mind that normal ECGs may not be surprising [156], our results report T wave inversion as the most common ECG abnormality, followed by ST-segment elevation. However, some studies suggest a widespread pattern in ECG alterations, limiting the ability to reach a definite localization [155, 156]. QT prolongation and transient Q waves were among other less frequent findings [155]. However, ECG alone cannot confirm the diagnosis, proposing the need for further evaluation [157].

Almost all patients had a reduced ejection fraction (EF) in the initial echocardiography study. We found that most patients survived and achieved a normal EF within 18 days. However, reduced LVEF is associated with an increased mortality risk [158]. Another study revealed that despite the recovery of LVEF, TCM has in-hospital mortality comparable to that of acute ST-segment elevation myocardial infarction [159].

There were limitations to this study. Despite our efforts to establish realistic and result-orientated selection criteria, the inherent variability in the methodology and reporting system of case-report studies may contribute to heterogeneity and adversely affect the generalizability of the findings. This also resulted in a considerable amount of missing or inconclusive data such as race, detailed past medical and familial history, clinical presentation, physical examination (specifically vital signs), and pharmacological therapeutic measures, which are points to address in future endeavors.

In conclusion, this study reviewed the association of TCM and autoimmune disorders. There were similarities in age, sex, and other aspects to classic TCM. The study suggests that autoimmune disease and TCM can coexist, and physicians should always consider TCM as a differential diagnosis of ACS if no obstructive coronary artery disease is noted. Further studies are needed, requiring precise reporting of future cases to perceive the role of autoimmunity in developing TCM.

## Figures and Tables

**Table 1:** Demographic data of 155 patients with Takotsubo cardiomyopathy associated with an autoimmune disease.

	Value
Gender	Frequency (%)
Male	25 (16.1%)
Female	130 (83.9%)
Total	155 (100%)
Age	Mean (SD)
Male	53.4 (22.9)
Female	56.3 (17.7)
Total	55.8 (18.6)
Race	Frequency (%)
Not reported	112 (71.3%)
Reported	45 (28.7%)
Caucasian	13 (29.5%)
African-American	10 (22.7%)
White	8 (18.2%)
Japanese	4 (9.1%)
Black	2 (4.5%)
Hispanic	2 (4.5%)
Chinese	2 (4.5%)
Asian	1 (2.3%)
Greek	1 (2.3%)
Korean	1 (2.3%)

**Table 2:** Background diseases of 155 patients with Takotsubo cardiomyopathy associated with an autoimmune disease, *n* (%).

	Male (*n* = 25)	Female (*n* = 130)	Total (*n* = 155)
Past medical history			
Not reported	13 (52.0%)	41 (31.5%)	55 (35.0%)
Reported	12 (48.0%)	89 (68.5%)	102 (65.0%)
Overall			
CVD	7 (28.0%)	37 (28.5%)	44 (28.0%)
AI	5 (20.0%)	75 (57.7%)	81 (51.5%)
Other diseases	7 (28.0%)	52 (40%)	60 (38.2%)
Case by case			
Isolated CVD	3 (12.0%)	4 (3.1%)	7 (4.4%)
Isolated AI	1 (4.0%)	23 (17.7%)	24 (15.2%)
Other diseases (only)	3 (12.0%)	9 (6.9%)	12 (7.6%)
CVD and AI	1 (4.0%)	10 (7.7%)	11 (7.0%)
CVD and other diseases	1 (4.0%)	1 (0.8%)	2 (1.2%)
AI and other diseases	1 (4.0%)	20 (15.4%)	22 (14.0%)
CVD and AI and other diseases	2 (8.0%)	22 (16.9%)	24 (15.2%)

CVD: cardiovascular disease, AI: autoimmune diseases.

**Table 3:** Clinical presentation of 155 patients with Takotsubo cardiomyopathy associated with an autoimmune disease, *n* (%).

Variable	Frequency (%)
Chief complaint	
Not reported	50 (32.3%)
Dyspnea	73 (46.9%)
Chest pain	55 (35.3%)
Palpitation	7 (4.4%)
Cyanosis	2 (1.2%)
Syncope	12 (7.7%)
Cardiopulmonary arrest	4 (2.5%)
Number of chief complaints	
Only one	65 (41.9%)
Two	32 (20.4%)
Three and more	8 (5.1%)
Abnormal vital sign	
Not reported	73 (47.1%)
At least one abnormal vital sign	82 (52.9%)
Tachycardia	37 (23.6%)
Bradycardia	3 (1.9%)
Hypertension	19 (12.2%)
Hypotension	33 (21%)
Fever	16 (10%)
Hypothermia	4 (2.5%)
Tachypnea	17 (10.7%)
Bradypnea	—
Abnormal cardiovascular exam	
Not reported	134 (86.5%)
Abnormal exam	21 (13.5%)
Abnormal CC, VS, PE	
No abnormality reported	29 (18.7%)
At least one abnormal finding	126 (81.3%)

**Table 4:** Autoimmune diseases of 155 patients with Takotsubo cardiomyopathy in descending order, *n* (%).

Autoimmune disease	*N* (%)
Myasthenia gravis	29 (18.8)
Systemic lupus erythematosus	22 (14.2)
Multiple sclerosis	18 (11.6)
Guillain–Barre syndrome	15 (9.7)
Grave's disease	12 (7.8)
Rheumatic arthritis	8 (5.2)
Autoimmune thyroiditis	5 (3.2)
Autoimmune poly-glandular syndrome type II	5 (3.2)
Vasculitis (ANCA-associated, IBD-associated, SLE vasculitis, cryoglobulinemic)	5 (3.2)
Inflammatory bowel disease (Crohn's, ulcerative colitis)	5 (3.2)
Autoimmune hepatitis	4 (2.5)
Arteritis (temporal, psoriatic, Takayasu)	3 (1.9)
Connective tissue disease	3 (1.9)
Polymyalgia rheumatica	3 (1.9)
Addison disease	2 (1.3)
Autoimmune limbic encephalitis	2 (1.3)
Rheumatic fever or rheumatic heart disease	2 (1.3)
Scleroderma and systemic sclerosis	2 (1.3)
Sjogren syndrome	2 (1.3)
Ankylosing spondylitis	1 (0.6)
Amyloidosis	1 (0.6)
Antiphospholipid syndrome	1 (0.6)
Autoimmune hemolytic anemia	1 (0.6)
Autoimmune hypothyroidism	1 (0.6)
Berger's disease	1 (0.6)
Celiac disease	1 (0.6)
Chronic arthritis	1 (0.6)
Dressler's syndrome	1 (0.6)
Henoch-Schönlein purpura	1 (0.6)
Immune mediated myocarditis	1 (0.6)
Juvenile rheumatic arthritis	1 (0.6)
Microscopic poly-angitis	1 (0.6)
Neuromyelitis optica	1 (0.6)
Necrotizing myopathy	1 (0.6)
Polyarteritis nodosa	1 (0.6)
Poly-myositis	1 (0.6)
Pernicious anemia	1 (0.6)
Primary biliary cholangitis	1 (0.6)
Primary sclerosing cholangitis	1 (0.6)
Sarcoidosis	1 (0.6)

**Table 5:** Echocardiography findings in 155 patients with Takotsubo cardiomyopathy associated with autoimmune disease, *n* (%).

	Male (*n* = 25)	Female (*n* = 130)	Total (*n* = 155)
First EF (EF1)			
Not reported	12 (38%)	29 (22.3%)	41 (26.5%)
Reported	13 (52%)	101 (77.7%)	114 (73.5%)
Reduced EF1	13 (52%)	100 (76.9%)	113 (72.9%)
Normal EF1	0 (0%)	1 (0.8%)	1 (0.6%)
Mean EF1 (%)	28.1 ± 10.7	29.8 ± 9	29.6 ± 9.2
Recovery EF (EF2)			
Not reported	11 (44.0%)	34 (26.2%)	45 (29%)
Reported EF2	14 (56.0%)	96 (73.8%)	110 (71%)
Reduced EF2	0 (0%)	7 (5.4%)	7 (4.5%)
Normal EF2	14 (56.0%)	89 (68.5%)	103 (66.5%)
Mean EF2 ± SD (%)	63.2 ± 6.6	58.3 ± 9	58.9 ± 8.8
EF recovery time			
Not applicable	9 (5.7%)	26 (20%)	35 (22.6%)
Reported	16 (10.1%)	104 (80%)	120 (77.4%)
Recovery time in days, median (IQR)	36.00 (14.00–82.50)	18.50 (8.50–42.00)	18.50 (10.00–42)

EF: ejection fraction.

## Data Availability

The authors confirm that the data supporting the findings of this study are available within the article and its supplementary materials.

## Supplementary Materials

Supplementary Materials Supplementary Material 1: Search strategy. Supplementary Material 2 (Table): A summary of data collected from all manuscripts included in this review.
