# Supplementary material for: Takotsubo Cardiomyopathy and Autoimmune Disorders: A Systematic Scoping Review of Published Cases
Source: Int J Clin Pract. 2024 Feb 20;2024:7259200. doi: 10.1155/2024/7259200 (PMC10898954; doi:10.1155/2024/7259200)
Supplement: Supplementary Materials — Supplementary Material 1: Search strategy. Supplementary Material 2 (Table): A summary of data collected from all manuscripts included in this review. [file 7259200.f1.docx]

Supplementary Material

Takotsubo cardiomyopathy and autoimmune disorders: A systematic scoping review of published cases

Mohsen Farjoud Kouhanjani^1,2,3^, Seyed Ali Hosseini^1,3^, Seyedeh Maryam Mousavi^3^, Zahra Noroozi^3^, Paniz Sadeghi^3^, Armita Jokar-Derisi^4^, Mohammad Saleh Jamshidi Mouselou^4^, Meysam Ahmadi^3^, Armin Attar^5*^

*** Correspondence:** Armin Attar, M.D., Ph.D. attarar@sums.ac.ir

# Search Strategy

## PubMed

(Takotsubo[tiab] OR Tako-tsubo[tiab] OR “Tako tsubo”[tiab] OR “Broken Heart Syndrome”[tiab] OR “Apical Ballooning Syndrome”[tiab] OR “Stress Cardiomyopathy”[tiab] OR “catecholamine cardiotoxicity”[tiab] OR “Apical ballooning cardiomyopathy”[tiab]) AND (Rheumatic[tiab] OR Rheumatoid[tiab] OR Rheumatology[tiab] OR “Collagen Diseases”[tiab] OR “Connective Tissue Disease”[tiab] OR lupus[tiab] OR Arthritis[tiab] OR Arthropathy[tiab] OR Arteritis[tiab] OR Spondylitis[tiab] OR Spondylarthropathy[tiab] OR Sclerosis[tiab] OR systemic[tiab] OR vasculitis[tiab] OR gout[tiab] OR Myalgia[tiab] OR Myositis[tiab] OR Myositides[tiab] OR “Inflammatory Muscle Disease”[tiab] OR “Inflammatory Myopathy”[tiab] OR “Inflammatory Muscle Disease”[tiab] OR “Inflammatory Myopathies”[tiab] OR behcet[tiab] OR Behçet[tiab] OR “Old Silk Route Disease”[tiab] OR “Triple Symptom Complex”[tiab] OR “Triple-Symptom Complex”[tiab] OR Nodosa[tiab] OR Chondritis[tiab] OR Tietze[tiab] OR Ankylosing[tiab] OR Fibromyalgia[tiab] OR “Muscular Rheumatism”[tiab] OR Kawasaki[tiab] OR “Mucocutaneous Lymph Node Syndrome”[tiab] OR joint[tiab] OR Psoriasis[tiab] OR psoriatic[tiab] OR Pustulosis[tiab] OR Autoimmune[tiab] OR Immune[tiab] OR Immunology[tiab] OR Immunologic[tiab] OR Sjogren[tiab] OR “Keratoconjunctivitis sicca”[tiab] OR Inflammation[tiab] OR Inflammatory[tiab] OR “multiple sclerosis”[tiab] OR “Disseminated Sclerosis”[tiab] OR “Optic Neuritis”[tiab] OR “Neuromyelitis Optica”[tiab] OR Devic[tiab] OR “Myasthenia Gravis”[tiab] OR “Guillain Barre”[tiab] OR “Guillaine-Barre”[tiab] OR “Guillaine Barre”[tiab] OR “Guillain-Barré”[tiab] OR “Guillain Barré”[tiab] OR “Autoimmune Neuropathy”[tiab] OR “Miller Fisher”[tiab] OR “Miller-fisher”[tiab] OR “transverse myelitis”[tiab] OR “Autoimmune Thyroiditis”[tiab] OR Hashimoto[tiab] OR Graves[tiab] OR Basedow[tiab] OR “autoimmune epilepsy”[tiab] OR “Inflammatory bowel disease”[tiab] OR Crohn[tiab] OR “Ulcerative colitis”[tiab] OR CIDP[tiab] OR “Chronic inflammatory demyelinating polyradiculoneuropathy”[tiab] OR Diabetes[tiab] OR Vitiligo[tiab] OR “Pernicious Anemia”[tiab] OR Addison[tiab] OR “Atrophic gastritis”[tiab] OR celiac[tiab] OR “Gluten enteropathy”[tiab] OR Sprue[tiab] OR Myocarditis[tiab] OR Pericarditis[tiab] OR Autoantibodies[tiab] OR Autoantibody[tiab] OR Nephritis[tiab] OR Glomerulonephritis[tiab] OR “Autoimmune Hepatitis”[tiab] OR Hypersensitivity[tiab] OR allergy[tiab] OR anaphylaxis[tiab] OR “multisystem inflammatory syndrome in children”[tiab] OR MISC[tiab] OR “MIS-C”[tiab] OR Angioedema[tiab] OR Dermatitis[tiab] OR Pemphigoid[tiab] OR Pemphigus[tiab] OR “Primary Biliary Cirrhosis”[tiab] OR “Primary Sclerosing Cholangitis”[tiab] OR Antiphospholipid[tiab] OR “Hughes Syndrome”[tiab] OR Raynaud[tiab] OR “CREST syndrome”[tiab] OR Scleroderma[tiab] OR “Cogan syndrome”[tiab] OR “Thromboangitis Obliterans”[tiab] OR Buerger[tiab] OR “Autoimmune Hemolytic Anemia”[tiab] OR “Immune Thrombocytopenic Purpura”[tiab] OR “Idiopathic Thrombocytopenic Purpura”[tiab] OR “Werlhof disease”[tiab] OR “Rheumatic Fever”[tiab] OR Henoch[tiab] OR “Wissler syndrome”[tiab] OR “lichen planus”[tiab] OR Takayasu[tiab])

## Embase

(Takotsubo:ti,ab,kw OR Tako-tsubo:ti,ab,kw OR ‘Tako tsubo’:ti,ab,kw OR ‘Broken Heart Syndrome’:ti,ab,kw OR ‘Apical Ballooning Syndrome’:ti,ab,kw OR ‘Stress Cardiomyopathy’:ti,ab,kw OR ‘catecholamine cardiotoxicity’:ti,ab,kw OR ‘Apical ballooning cardiomyopathy’:ti,ab,kw) AND (Rheumatic:ti,ab,kw OR Rheumatoid:ti,ab,kw OR Rheumatology:ti,ab,kw OR ‘Collagen Diseases’:ti,ab,kw OR ‘Connective Tissue Disease’:ti,ab,kw OR lupus:ti,ab,kw OR Arthritis:ti,ab,kw OR Arthropathy:ti,ab,kw OR Arteritis:ti,ab,kw OR Spondylitis:ti,ab,kw OR Spondylarthropathy:ti,ab,kw OR Sclerosis:ti,ab,kw OR systemic:ti,ab,kw OR vasculitis:ti,ab,kw OR gout:ti,ab,kw OR Myalgia:ti,ab,kw OR Myositis:ti,ab,kw OR Myositides:ti,ab,kw OR ‘Inflammatory Muscle Disease’:ti,ab,kw OR ‘Inflammatory Myopathy’:ti,ab,kw OR ‘Inflammatory Muscle Disease’:ti,ab,kw OR ‘Inflammatory Myopathies’:ti,ab,kw OR behcet:ti,ab,kw OR Behçet:ti,ab,kw OR ‘Old Silk Route Disease’:ti,ab,kw OR ‘Triple Symptom Complex’:ti,ab,kw OR ‘Triple-Symptom Complex’:ti,ab,kw OR Nodosa:ti,ab,kw OR Chrondritis:ti,ab,kw OR Tietze:ti,ab,kw OR Ankylosing:ti,ab,kw OR Fibromyalgia:ti,ab,kw OR ‘Muscular Rheumatism’:ti,ab,kw OR Kawasaki:ti,ab,kw OR ‘Mucocutaneous Lymph Node Syndrome’:ti,ab,kw OR joint:ti,ab,kw OR Psoriasis:ti,ab,kw OR psoriatic:ti,ab,kw OR Pustulosis:ti,ab,kw OR Autoimmune:ti,ab,kw OR Immune:ti,ab,kw OR Immunology:ti,ab,kw OR Immunologic:ti,ab,kw OR Sjogren:ti,ab,kw OR ‘Keratoconjunctivitis sicca’:ti,ab,kw OR Inflammation:ti,ab,kw OR Inflammatory:ti,ab,kw OR ‘multiple sclerosis’:ti,ab,kw OR ‘Disseminated Sclerosis’:ti,ab,kw OR ‘Optic Neuritis’:ti,ab,kw OR ‘Neuromyelitis Optica’:ti,ab,kw OR Devic:ti,ab,kw OR ‘Myasthenia Gravis’:ti,ab,kw OR ‘Guillain Barre’:ti,ab,kw OR ‘Guillaine-Barre’:ti,ab,kw OR ‘Guillaine Barre’:ti,ab,kw OR ‘Guillain-Barré’:ti,ab,kw OR ‘Guillain Barré’:ti,ab,kw OR ‘Autoimmune Neuropathy’:ti,ab,kw OR ‘Miller Fisher’:ti,ab,kw OR ‘Miller-fisher’:ti,ab,kw OR ‘transverse myelitis’:ti,ab,kw OR ‘Autoimmune Thyroiditis’:ti,ab,kw OR Hashimoto:ti,ab,kw OR Graves:ti,ab,kw OR Basedow:ti,ab,kw OR ‘autoimmune epilepsy’:ti,ab,kw OR ‘Inflammatory bowel disease’:ti,ab,kw OR Crohn:ti,ab,kw OR ‘Ulcerative colitis’:ti,ab,kw OR CIDP:ti,ab,kw OR ‘Chronic inflammatory demyelinating polyradiculoneuropathy’:ti,ab,kw OR Diabetes:ti,ab,kw OR Vitiligo:ti,ab,kw OR ‘Pernicious Anemia’:ti,ab,kw OR Addison:ti,ab,kw OR ‘Atrophic gastritis’:ti,ab,kw OR celiac:ti,ab,kw OR ‘Gluten enteropathy’:ti,ab,kw OR Sprue:ti,ab,kw OR Myocarditis:ti,ab,kw OR Pericarditis:ti,ab,kw OR Autoantibodies:ti,ab,kw OR Autoantibody:ti,ab,kw OR Nephritis:ti,ab,kw OR Glomerulonephritis:ti,ab,kw OR ‘Autoimmune Hepatitis’:ti,ab,kw OR Hypersensitivity:ti,ab,kw OR allergy:ti,ab,kw OR anaphylaxis:ti,ab,kw OR ‘multisystem inflammatory syndrome in children’:ti,ab,kw OR MISC:ti,ab,kw OR ‘MIS-C’:ti,ab,kw OR Angioedema:ti,ab,kw OR Dermatitis:ti,ab,kw OR Pemphigoid:ti,ab,kw OR Pemphigus:ti,ab,kw OR ‘Primary Biliary Cirrhosis’:ti,ab,kw OR ‘Primary Sclerosing Cholangitis’:ti,ab,kw OR Antiphospholipid:ti,ab,kw OR ‘Hughes Syndrome’:ti,ab,kw OR Raynaud:ti,ab,kw OR ‘CREST syndrome’:ti,ab,kw OR Scleroderma:ti,ab,kw OR ‘Cogan syndrome’:ti,ab,kw OR ‘Thromboangitis Obliterans’:ti,ab,kw OR Buerger:ti,ab,kw OR ‘Autoimmune Hemolytic Anemia’:ti,ab,kw OR ‘Immune Thrombocytopenic Purpura’:ti,ab,kw OR ‘Idiopathic Thrombocytopenic Purpura’:ti,ab,kw OR ‘Werlhof disease’:ti,ab,kw OR ‘Rheumatic Fever’:ti,ab,kw OR Henoch:ti,ab,kw OR ‘Wissler syndrome’:ti,ab,kw OR ‘lichen planus’:ti,ab,kw OR Takayasu:ti,ab,kw)

## Web of Science

TS=(Takotsubo OR Tako-tsubo OR “Tako tsubo” OR “Broken Heart Syndrome” OR “Apical Ballooning Syndrome” OR “Stress Cardiomyopathy” OR “catecholamine cardiotoxicity” OR “Apical ballooning cardiomyopathy”) AND TS=(Rheumatic OR Rheumatoid OR Rheumatology OR “Collagen Diseases” OR “Connective Tissue Disease” OR lupus OR Arthritis OR Arthropathy OR Arteritis OR Spondylitis OR Spondylarthropathy OR Sclerosis OR systemic OR vasculitis OR gout OR Myalgia OR Myositis OR Myositides OR “Inflammatory Muscle Disease” OR “Inflammatory Myopathy” OR “Inflammatory Muscle Disease” OR “Inflammatory Myopathies” OR behcet OR Behçet OR “Old Silk Route Disease” OR “Triple Symptom Complex” OR “Triple-Symptom Complex” OR Nodosa OR Chondritis OR Tietze OR Ankylosing OR Fibromyalgia OR “Muscular Rheumatism” OR Kawasaki OR “Mucocutaneous Lymph Node Syndrome” OR joint OR Psoriasis OR psoriatic OR Pustulosis OR Autoimmune OR Immune OR Immunology OR Immunologic OR Sjogren OR “Keratoconjunctivitis sicca” OR Inflammation OR Inflammatory OR “multiple sclerosis” OR “Disseminated Sclerosis” OR “Optic Neuritis” OR “Neuromyelitis Optica” OR Devic OR “Myasthenia Gravis” OR “Guillain Barre” OR “Guillaine-Barre” OR “Guillaine Barre” OR “Guillain-Barré” OR “Guillain Barré” OR “Autoimmune Neuropathy” OR “Miller Fisher” OR “Miller-fisher” OR “transverse myelitis” OR “Autoimmune Thyroiditis” OR Hashimoto OR Graves OR Basedow OR “autoimmune epilepsy” OR “Inflammatory bowel disease” OR Crohn OR “Ulcerative colitis” OR CIDP OR “Chronic inflammatory demyelinating polyradiculoneuropathy” OR Diabetes OR Vitiligo OR “Pernicious Anemia” OR Addison OR “Atrophic gastritis” OR celiac OR “Gluten enteropathy” OR Sprue OR Myocarditis OR Pericarditis OR Autoantibodies OR Autoantibody OR Nephritis OR Glomerulonephritis OR “Autoimmune Hepatitis” OR Hypersensitivity OR allergy OR anaphylaxis OR “multisystem inflammatory syndrome in children” OR MISC OR “MIS-C” OR Angioedema OR Dermatitis OR Pemphigoid OR Pemphigus OR “Primary Biliary Cirrhosis” OR “Primary Sclerosing Cholangitis” OR Antiphospholipid OR “Hughes Syndrome” OR Raynaud OR “CREST syndrome” OR Scleroderma OR “Cogan syndrome” OR “Thromboangitis Obliterans” OR Buerger OR “Autoimmune Hemolytic Anemia” OR “Immune Thrombocytopenic Purpura” OR “Idiopathic Thrombocytopenic Purpura” OR “Werlhof disease” OR “Rheumatic Fever” OR Henoch OR “Wissler syndrome” OR “lichen planus” OR Takayasu)

# Supplementary Table. Data collected from all manuscripts included in this review

| **Intext citation** |  | **Age** | **Gender** | **Race** | **Country** | **PmHx** | **CC** | **VS** | **CV exam** | **AI Disease** | **AI marker** | **Trigger** | **Lab data** | **Trop** | **ECG** | **Angio** | **1^st^ EF** | **2^nd^ EF** | **Outcome** |
| --- | --- | --- | --- | --- | --- | --- | --- | --- | --- | --- | --- | --- | --- | --- | --- | --- | --- | --- | --- |
| 116 | A case of persistent apical ballooning complicated by apical thrombus in takotsubo cardiomyopathy of systemic lupus erythematosus patient (1) | 63 | F | KOREAN | KOREA | CVD,AI | Dyspnea | Tachycardia, Tachypnea | AbN | SLE | Neg | NR | CKMB | E | STE | N | 42 | 50 | D |
| 120 | A case of reverse takotsubo cardiomyopathy caused by an eating disorder(2) | 67 | F | NR | USA | AI,Other | Dyspnea, CP,Syncope | Tachycardia | NR | Crohn 's disease | NR | Psy,Phy | BNP | E | Other,T-invertion | N | 25 | NR | S |
| 94 | A case of takotsubo cardiomyopathy leading to the diagnosis of myasthenia gravis (3) | 52 | F | NR | Japan | Other | Dyspnea | Tachycardia, Hypertension | AbN | MG | NR | Phy | NR | E | STE | N | 45 | 57 | S |
| 105 | A case of Takotsubo syndrome during a multiple sclerosis brainstem relapse (4) | 45 | F | Caucasian | Italy | AI | Dyspnea,CP | Tachycardia | NR | MS | NR | NR | NR | E | STE,T-invertion | N | 25 | NR | S |
| 24 | A Case Report of Recurrent Takotsubo Cardiomyopathy in a Patient during Myasthenia Crisis ( first episode) (5) | 69 | F | Caucasian | USA | CVD,AI,Other | CP,Palplitation | NR | AbN | MG , Graves disease | P | Phy | NR | E | STE,Other,T-invertion | N | 30 | N | S |
| 24 | A Case Report of Recurrent Takotsubo Cardiomyopathy in a Patient during Myasthenia Crisis 2(second episode) (5) | 69 | F | Caucasian | USA | CVD,AI,Other | CP | NR | NR | MG , Graves disease | Neg | Phy | NR | E | T-invertion | NR | NR | 60 | S |
| 110 | A Myasthenic Crisis Complicated by a Takotsubo Cardiomyopathy (6) | 70 | F | NR | Spain | CVD,AI,Other | Dyspnea | Hypotension | NR | thymoma-positive MG | NR | Phy | NR | E | Other,T-invertion | NR | 30-35 | 65 | S |
| 89 | A rare association of systemic lupus erythematosus, morbid obesity and Takotsubo syndrome (7) | 68 | F | white | Brazil | AI,Other | Dyspnea,CP,  Cyanosis | NR | NR | SLE | NR | Phy | NR | N | N | N | NR | NR | S |
| 29 | A rare stress cardiomyopathy in a patient with Guillain-Barré syndrome (8) | 36 | F | NR | Netherlands | AI | Dyspnea | Tachycardia, Hypotension,Fever | NR | GBS | NR | Phy | BNP | E | Other | NR | 10 | N | S |
| 68 | A simple procedure in a complex patient: Perioperative takotsubo cardiomyopathy (9) | 43 | F | NR | Australia | AI,Other | Cardio-pulmonary arrest | Hypotension | AbN | autoimmune hepatitis | NR | Psy,Phy | BNP | E | Other,T-invertion | NR | 33 | 71 | S |
| 78 | A tongue lesion as a sign of a systemic disease (10) | 77 | F | NR | Greece | CVD,AI  ,Other | Dyspnea | Tachycardia | AbN | questionable SLE and al amyloidosis | P | NR | BNP,CKMB | Borderline | NR | N | NR | NR | D |
| 33 | Acute biopsy-proven lymphocytic myocarditis mimicking Takotsubo cardiomyopathy (11) | 68 | F | NR | Italy | NR | Dyspnea,CP | Tachycardia | NR | immune-mediated myocarditis | P | Phy | NR | E | STE,T-invertion | N | 23 | N | S |
| 93 | Altered mental status in an elderly woman with concurrent takotsubo syndrome and polymyalgia rheumatica: a case of treatable geriatric delirium (12) | 80 | F | black | USA | CVD,Other | NR | Fever, Hypotension, Tachypnea | NR | polymyalgia rheumatica | Neg | Psy | ESR, CRP | N | Other | NR | 20 | N | S |
| 90 | An uncommon first manifestation of multiple sclerosis: Tako-Tsubo cardiomyopathy(13) | 23 | M | NR | Spain | NR | Dyspnea | Hypertension, Tachypnea | NR | multiple sclerosis | P | NR | NR | E | STD | NR | 35 | 62 | S |
| 66 | An Unfortunate Case of Takotsubo Cardiomyopathy During Plasmapheresis for Myasthenia Crisis (14) | 72 | M | NR | USA | CVD | NR | Hypotension, Tachypnea,Tachycardia | NR | MG | NR | Phy | NR | E | STE | NR | 12 | NR | D |
| 43 | An Unusual Cardiomyopathy after Phy Stress in a Child (15) | 4 | F | NR | Italy | AI | NR | Tachycardia, Tachypnea ,Fever | AbN | celiac disease | Neg | Phy | CRP | E | STE,STD | N | 35 | 47 | S |
| 31 | An Unusual Case of Shock Following an Elective Caesarean Delivery (16) | 31 | F | NR | Australia | AI,Other | CP | Tachycardia | NR | polyglandular autoimmune syndrome type2 , Hashimoto’s thyroiditis ,celiac disease | NR | Phy | BNP | E | STD.T-invertion | NR | 20 | 50 | S |
| 50 | Apical Ballooning Resulting from Limbic Encephalitis (17) | 73 | F | NR | USA | AI | NR | NR | NR | limbic encephalitis | NR | Phy | NR | E | T-invertion | N | NR | NR | S |
| 102 | Biventricular Takotsubo cardiomyopathy in graves hyperthyroidism (18) | 36 | F | African-American | USA | CVD,AI | NR | NR | NR | GRAVES | NR | Phy | NR | Not checked | T-invertion | N | 25 | N | S |
| 129 | Biventricular Tako-Tsubo cardiomyopathy: Usefulness of 2D speckle tracking strain echocardiography (19) | 55 | F | NR | Italy | AI,Other | NR | NR | NR | Multiple sclerosis | NR | Phy | NR | E | Other | N | 30 | N | S |
| 123 | Broken heart syndrome during myasthenic crisis (20) | 42 | F | NR | USA | Other | Dyspnea | NR | NR | MG | NR | Phy | NR | E | NR | NR | 15 | 45 | S |
| 21 | Broken heart syndrome in myasthenia gravis (21) | 77 | M | NR | USA | NR | Dyspnea | Tachypea | NR | MG | P | Phy | CKMB | E | STE | N | 20 | 60 | S |
| 106 | Cardiogenic shock due to Takotsubo cardiomyopathy following thyroidectomy(22) | 63 | F | NR | Sri Lanka | NR | Dyspnea | Hypotension, Tachycardia | AbN | autoimmune hemolytic anemia (IgG),  autoimmune thyroiditis | NR | Phy | NR | E | T-invertion | N | 35 | N | S |
| 124 | Cardiogenic shock following cardiac tamponade and Takotsubo in COVID-19 (23) | 42 | F | NR | USA | AI | NR | Tachycardia- Fever | NR | Crohn’s disease & GBS | NR | Phy | BNP | E | Other | N | 20 | NR | D |
| 62 | Case of Takotsubo cardiomyopathy after tooth extraction - Unusual trigger of a rare syndrome (24) | 75 | F | NR | Germany | CVD,AI,Other | Dyspnea,CP | NR | NR | Chronic arthritis | NR | Phy | NR | E | T-invertion | N | 30-35 | 55 | S |
| 119 | Coincidence of apical ballooning syndrome (tako-tsubo/stress cardiomyopathy) and posterior reversible encephalopathy syndrome: Potential common substrate and pathophysiology? Case1 (25) | 83 | F | NR | USA | CVD,AI,Other | Dyspnea | Hypertension | NR | Guillain–Barré syndrome | NR | Phy | NR | E | STE,T-invertion | NR | 35 | 67 | S |
| 119 | Coincidence of apical ballooning syndrome (tako-tsubo/stress cardiomyopathy) and posterior reversible encephalopathy syndrome: Potential common substrate and pathophysiology? Case 2 (25) | 60 | F | NR | USA | CVD,AI,Other | Dyspnea,CP | Hypertension | NR | SLE mesenteric vasculitis, treated with steroid | NR | Phy | NR | E | T-invertion | NR | 25 | 50 | NR |
| 119 | Coincidence of apical ballooning syndrome (tako-tsubo/stress cardiomyopathy) and posterior reversible encephalopathy syndrome: Potential common substrate and pathophysiology? Case 3 (25) | 47 | F | NR | USA | CVD,AI,Other | intubated | Hypertension | NR | Sjogren syndrome on steroids. | NR | Phy | NR | E | T-invertion | NR | 25 | 55 | S |
| 119 | Coincidence of apical ballooning syndrome (tako-tsubo/stress cardiomyopathy) and posterior reversible encephalopathy syndrome: Potential common substrate and pathophysiology? Case 4 (25) | 64 | F | NR | USA | CVD,AI,Other | Dyspnea,CP | NR | NR | Henoch-Sch€onlein purpura and IgA nephropathy | NR | Phy | NR | E | STE,T-invertion | NR | 40 | 60 | NR |
| 63 | Early-onset cardiomyopathy after pacemaker implanted in a preterm infant with congenital complete heart block and anti-ro/ssa antibodies (26) | Infant  (at birth) | F | NR | USA | NR | NR | Bradycardia | NR | Congenital Sjogren dx (Anti-Ro/SSA)Congenital Sjogren(Anti-Ro/SSA) | P | Phy | NR | N | Other | NR | 39 | 60 | S |
| 23 | Electrocardiographic ST-segment elevation: Takotsubo cardiomyopathy versus ST-segment elevation myocardial infarction-A case series (27) | 64 | F | NR | USA | CVD,AI | CP,Dyspnea | NR | NR | rheumatic fever | NR | Phy | NR | E | STE | N | 35 | NR | S |
| 122 | Fulminant Guillain-Barre syndrome with Takotsubo cardiomyopathy: Report of an autopsied case (28) | 77 | M | japanese | Japan | NR | Dyspnea | Fever, Hypotension | NR | fulminant GBS | NR | Phy | BNP | Not checked | T-invertion | N | NR | NR | D |
| 109 | Guillain-Barré syndrome and catecholamine therapy. A potential risk for developing takotsubo cardiomyopathy? (29) | 59 | F | NR | Germany | NR | Syncope | NR | NR | Guillain–Barré syndrome | NR | Phy | CKMB | E | STE | N | 25 | NR | NR |
| 67 | Guillain-Barré syndrome complicated by takotsubo cardiomyopathy: An under-recognised association (30) | 41 | F | NR | UK | NR | Dyspnea,CP | NR | NR | Guillain-Barré syndrome | NR | Phy | NR | Not checked | STE/STD | N | NR | 46 | S |
| 71 | Haemophagocytic lymphohistiocytosis (HLH)-associated stress cardiomyopathy secondary to autoimmune conditions successfully treated with anakinra,case1 (31) | 39 | M | white Irish | Ireland | AI | NR | Hypotension, Fever | NR | Still’s disease(juvenile RA) | Neg | Phy | NR | E | Other | NR | 40 | N | S |
| 71 | Haemophagocytic lymphohistiocytosis (HLH)-associated stress cardiomyopathy secondary to autoimmune conditions successfully treated with anakinra,case2 (31) | 32 | F | white Irish | Ireland | AI | NR | Hypotension, Fever | NR | SLE | NR | Phy | NR | E | Other | NR | 10 | 55 | S |
| 47 | Hypertension, tachycardia, and reversible cardiomyopathy temporally associated with milnacipran use (32) | 42 | F | NR | Belgium/USA | NR | CP | Hypertension | AbN | undefined connective-tissue disease | P | Phy | NR | Not checked | Other/T-invertion | NR | 30 | 2 | S |
| 117 | Is the spiked helmet sign the manifestation of long QT syndrome? (33) | 47 | F | NR | Hungary | AI,Other | Dyspnea,CP,Syncope | NR | NR | Buerger’s disease | NR | Psy | NR | E | STE,Other | N | decreased | NR | S |
| 113 | Isolated Left Ventricular Basal Ballooning Phenotype of Transient Cardiomyopathy in Young Women (34) | 32 | F | white | USA | NR | CP | NR | NR | MS | NR | Phy | NR | E | T-invertion | N | 45 | NR | S |
| 97 | Levosimendan: The Inotrope of Choice in Cardiogenic Shock Secondary to Takotsubo Cardiomyopathy? (35) | 80 | F | NR | Australia | AI | NR | NR | NR | myasthaenia gravis | NR | NR | NR | Not checked | T-invertion | N | 35-40 | NR | NR |
| 55 | Liver Transplantation Followed by Renal Transplantation on Extracorporeal Membrane Oxygenation: A Case Report (36) | 68 | F | NR | USA | CVD,AI,Other | Dyspnea,CP,Palplitation | NR | NR | autoimmune hepatitis | NR | NR | NR | Not checked | NR | NR | 10 | NR | S |
| 36 | Lupus myopericarditis as a preceding stressor for takotsubo cardiomyopathy (37) | 61 | F | African American | USA | CVD,AI | CP | NR | AbN | SLE | NR | Phy | CKMB | E | STE,Other,T-invertion | N | 25 | 52.5 | S |
| 17 | Multiple sclerosis broke my heart case 1 (38) | 18 | M | NR | france | NR | Dyspnea | Hypotension | NR | RRMS | NR | Phy | NR | E | STD | N | 15 | 74 | S |
| 17 | Multiple sclerosis broke my heart case 2(38) | 22 | M | NR | france | NR | Dyspnea,CP | hypothermia, Hypotension | NR | RRMS (relapsing- remitting multiple sclerosis) | NR | Phy | NR | E | T-invertion | N | 40 | 60 | S |
| 17 | Multiple sclerosis broke my heart case 3(38) | 16 | F | NR | france | AI | Dyspnea | Hypotension,hypothermia | NR | RRMS | NR | Phy | NR | E | STE | N | 35 | 60 | S |
| 17 | Multiple sclerosis broke my heart case 4(38) | 25 | F | NR | france | NR | Dyspnea | Hypotension | NR | RRMS | NR | Phy | NR | E | STE | N | 35 | 65 | S |
| 17 | Multiple sclerosis broke my heart case 5 (38) | 27 | F | NR | france | NR | Dyspnea | Hypotension | NR | RRMS | NR | Phy | NR | E | Other | N | 10 | 68 | S |
| 74 | Multiple sclerosis lesion in the medulla oblongata in a patient with takotsubo cardiomyopathy (39) | 30 | F | NR | Japan | AI | CP,Palplitation | Hypertension | NR | MS | NR | Phy | CKMB | N | STD.Other | N | NR | NR | S |
| 25 | Multiple sclerosis relapse presenting as an acute cardiomyopathy case 1 (40) | 52 | M | NR | USA | CVD | Dyspnea,CP | bradycardia | NR | MS | P | Phy | NR | E | STD | N | 35 | 65 | S |
| 25 | Multiple sclerosis relapse presenting as an acute cardiomyopathy case 2(40) | 57 | F | NR | USA | AI | Dyspnea,CP | NR | NR | MS | P | Phy | NR | E | Other | N | 30 | 73 | S |
| 75 | Myasthenic crisis-induced Takotsubo cardiomyopathy in an elderly man: A case report of an underestimated but deadly combination (41) | 81 | M | NR | Netherlands | Other | Dyspnea | Hypertension | NR | myasthenia gravis | P | Phy | NR | E | STE.T-invertion | N | NR | NR | D |
| 65 | Myocardial edema in Takotsubo syndrome mimicking apical hypertrophic cardiomyopathy: An insight into diagnosis by cardiovascular magnetic resonance (42) | 44 | M | NR | UK | CVD,AI | CP | NR | NR | ankylosing spondylitis | NR | NR | NR | E | STE/T-invertion | N | NR | 71 | S |
| 46 | Noncompaction and Takotsubo Syndrome in a Neuromuscular Disorder (43) | 68 | F | Caucasian | Austria | CVD | CP | NR | NR | Hashimoto thyroiditis | NR | Psy | NR | E | STE | N | NR | N | S |
| 48 | One thing leads to another: GBS complicated by PRES and takotsubo cardiomyopathy (44) | 82 | F | NR | USA | CVD | NR | tachypneic | NR | Guillane–Barre´ syndrome | NR | Phy | NR | E | STE | N | 30 | 67 | S |
| 58 | Onset of Takotsubo Syndrome during the Clinical Course of Anti-Neutrophil Cytoplasmic Antibody-Associated Vasculitis: A Case Report (45) | 78 | F | Japanese | Japan | Other | Dyspnea | Tachycardia, Hypertension | AbN | Anti-neutrophil cytoplasmic antibody (ANCA)-associated vasculitis | P | Psy,Phy | BNP | Not checked | Other/T-invertion | N | 20-25 | 63 | S |
| 92 | Pericarditis-complicated takotsubo cardiomyopathy in a patient with rheumatoid arthritis (46) | 64 | F | NR | Japan | AI | Dyspnea,CP | NR | NR | RA | NR | NR | CRP | E | STE,Other,T-invertion | N | NR | N | S |
| 14 | Polyglandular endocrine emergency: Lessons from a patient, which a book cannot teach(47) | 30 | F | NR | UK | AI | Cardiopulmonary arrest,Syncope | bradycardia,Hypotension,hypothermia | AbN | polyglandular autoimmune type 2 syndrome (Addison’s disease and type 1 diabetes) and coeliac disease | P | Phy | CRP | E | Other | N | NR | NR | S |
| 26 | Postoperative left ventricular apical ballooning: Transient takotsubo cardiomyopathy following orthotopic liver transplantation(48) | 51 | F | NR | Czech Republic | AI,Other | NR | Hypotension | NR | SLE and autoimmune hepatitis | NR | Phy | NR | E | T-invertion | NR | 20 | 50 | S |
| 79 | Post-procedural inverted Takotsubo cardiomyopathy(49) | 72 | F | NR | USA | AI | Dyspnea | NR | NR | rheumatoid arthritis | NR | NR | NR | E | STE or STD | NR | NR | NR | S |
| 16 | Pulmonary edema in myasthenic crisis (50) | 50 | F | NR | India | NR | Dyspnea | Tachycardia,Tachypnea, Hypotension | NR | myasthenia gravis | P | Phy | CKMB | N | Other | NR | 35 | 55 | S |
| 131 | Recurrent takotsubo cardiomyopathy precipitated by myasthenic crisis (51) | 64 | F | Chinese | Singapore | NR | Dyspnea,CP | NR | NR | myasthenic crisis | NR | NR | NR | E | N | N | 30 | N | S |
| 99 | Recurrent takotsubo cardiomyopathy related to recurrent thyrotoxicosis(52) | 55 | F | black | USA | CVD,AI,Other | Dyspnea,CP | Tachycardia | NR | GRAVES | NR | Psy | CKMB | E | Other | N | 30 | 75 | S |
| 45 | Respiratory insufficiency from myasthenia gravis and polymyositis due to malignant thymoma triggering Takotsubo syndrome(53) | 72 | F | NR | Austria | CVD | Cardiopulmonary arrest | NR | NR | MG, polymyositis | P | Psy | NR | E | Other | N | reduced | NR | D |
| 83 | Reverse takotsubo cardiomyopathy triggered by a multiple sclerosis relapse(54) | 30 | F | NR | Belgium | NR | Dyspnea,CP,Palplitation | Tachycardia | NR | MS | NR | Phy | NR | E | Other | NR | 20 | 50 | S |
| 101 | Reverse Takotsubo syndrome in a patient with diagnosed multiple sclerosis(55) | 43 | F | NR | Poland | AI | Dyspnea,CP | Tachycadia | NR | MS | NR | Psy | CKMB | E | STE,STD | N | NR | NR | S |
| 27 | Reversed takotsubo cardiomyopathy in a patient with new-onset multiple sclerosis: Cause, link, or just coincidence?(56) | 29 | M | NR | Netherlands | NR | CP | Hypertension | NR | MS | NR | Phy | CKMB | E | N,STD | N | NR | NR | S |
| 61 | Reversible Left Ventricular Dysfunction Associated with Guillain-Barré Syndrome:An Expression of Catecholamine Cardiotoxicity?(57) | 76 | F | NR | Japan | NR | NR | NR | NR | Guillain-Barré | NR | Phy | NR | Not checked | STE | N | 36 | NR | S |
| 98 | Reversible posterior leukoencephalopathy syndrome and takotsubo cardiomyopathy: The role of echocardiographic monitoring in the ICU(58) | 47 | F | FALSE | Greece | NR | NR | Hypertension | NR | Polyartritis Nodusa | NR | NR | NR | E | N | NR | NR | NR | NR |
| 121 | Reversible posterior leukoencephalopathy syndrome in p-ANCA-associated vasculitis(59) | 76 | F | NR | Japan | NR | Syncope | Fever | NR | isolated oculomotor neuropathy associated with pANCA-positive vasculitic syndrome | P | Phy | ESR,CRP | Not checked | NR | NR | NR | NR | D |
| 56 | Reversible Stress Cardiomyopathy in Guillain-Barre Syndrome: A Case Report(60) | 65 | F | Greek | Greece | Other | Dyspnea | Hypotension | NR | GBS | NR | Phy | BNP | E | Other/T-invertion | N | 20 | N | S |
| 35 | Role of cardiac magnetic resonance in the differential diagnosis of Takotsubo cardiomyopathy(61) | 72 | F | NR | Italy | CVD,AI,Other | NR | Hypotension | NR | psoriatic arthritis | NR | NR | NR | E | STE,Other | N | 25 | 38 | S |
| 130 | Sepsis-induced takotsubo syndrome in young premenopausal women: Two case reports(62) | 48 | F | NR | China | AI | Dyspnea | Tachycardia, Tachypnea, E BP -Fever | NR | mixed connective tissue diseas | NR | Phy | CRP,CKMB | E | T-invertion | NR | 30 | 50 | S |
| 60 | Severe respiratory failure and Torsades de Pointes induced by disopyramide in a patient with myasthenia gravis(63) | 63 | F | NR | Japan | CVD,AI | Dyspnea | NR | NR | Myasthenia Gravis | P | Phy | NR | Not checked | Other/T-invertion | NR | NR | NR | D |
| 95 | Severe sepsis caused by Aeromonas hydrophila in a patient using tocilizumab: A case report(64) | 72 | F | Japanese | Japan | AI | NR | Hypotension, Fever | NR | RA | NR | Phy | CRP | Not checked | STE | N | NR | N | S |
| 85 | Severe takotsubo cardiomyopathy following orthotopic liver transplantation: A case series(65) | 47 | F | NR | USA | AI | Dyspnea,CP | Hypotension | NR | primary biliary cholangitis and Sjrogrens/autoimmune hepatitis | NR | Phy | NR | N | NR | N | 15-20 | 60 | S |
| 41 | Severe transient left ventricular dysfunction in a patient with Legionella pneumophila pneumonia(66) | 51 | F | NR | Poland | AI,Other | Dyspnea | Tachycardia | NR | cryoglobulinemic vasculitis, Sjögren syndrome | NR | NR | ESR,CRP | E | Other | N | 25 | 60 | S |
| 28 | Severe transient left ventricular dysfunction induced by thyrotoxicosis(67) | 44 | F | NR | Netherlands | AI | Dyspnea,CP,Palplitation | tahycardia,Hypertension | NR | Graves disease | NR | Phy | CRP | E | N,T-invertion | N | REDUCED | 52 | S |
| 32 | Storm and STEMI: a case report of unexpected cardiac complications of thyrotoxicosis(68) | 23 | M | NR | USA | NR | NR | Tachycardia, Hypotension, Tachypnea, Fever | NR | Grave’s disease | P | Phy | NR | E | STE.Other | NR | 23 | 65 | S |
| 72 | Stress cardiomyopathy (takotsubo cardiomyopathy)(69) | 57 | F | white | USA | CVD,AI | CP | NR | NR | rheumatoid arthritis | NR | NR | NR | E | T-invertion | N | 40 | N | S |
| 39 | Stress cardiomyopathy (Takotsubo) following radioactive iodine therapy(70) | 55 | F | NR | UK | AI | Dyspnea,CP | NR | NR | Graves disease | NR | Phy | NR | E | STE,Other | N | 30-35 | 55 | S |
| 15 | Stress cardiomyopathy associated with area postrema syndrome as a presentation of neuromyelitis optica: Case report(71) | 30 | F | Asian | South Korea | NR | Dyspnea | NR | NR | neuromyelitis optica spectrum disorder with AQPPalplitation-IgG | P | NR | BNP,CKMB | E | STE | N | 13 | N | S |
| 112 | Stress cardiomyopathy associated with the first manifestation of multiple sclerosis: A case report(72) | 19 | M | NR | Germany | NR | Dyspnea | Hypertension | NR | MS | NR | NR | NR | E | N | N | NR | NR | S |
| 18 | Stress cardiomyopathy in dextrocardia with situs inversus and anomalous coronary arteries(73) | 53 | M | NR | Australia | CVD,AI,Other | Dyspnea,CP | Tachycardia, Tachypnea | AbN | polymyalgia rheumatica | NR | NR | NR | E | T-invertion | N | NR | NR | S |
| 54 | Stress Cardiomyopathy Precipitated by Withdrawal of Epoprostenol(74) | 67 | F | NR | USA | CVD,AI,Other | NR | Hypotension | AbN | scleroderma | NR | Phy | BNP | E | Other/T-invertion | N | 25-30 | 60 | S |
| 143 | Stress from myasthenic crisis triggers Takotsubo (broken heart) syndrome case 1(75) | 75 | F | NR | Austria | NR | NR | NR | NR | MG | NR | Phy | NR | Not checked | NR | NR | NR | NR | S |
| 143 | Stress from myasthenic crisis triggers Takotsubo (broken heart) syndrome case 2(75) | 83 | M | NR | Austria | NR | NR | NR | NR | MG | NR | Phy | NR | Not checked | NR | NR | NR | NR | S |
| 143 | Stress from myasthenic crisis triggers Takotsubo (broken heart) syndrome case 3(75) | 82 | F | NR | Austria | NR | NR | NR | NR | MG | NR | NR | NR | Not checked | NR | NR | NR | NR | NR |
| 143 | Stress from myasthenic crisis triggers Takotsubo (broken heart) syndrome case 4(75) | 80 | F | NR | Austria | NR | NR | NR | NR | MG, thymectomy | NR | NR | NR | Not checked | NR | NR | NR | NR | NR |
| 143 | Stress from myasthenic crisis triggers Takotsubo (broken heart) syndrome case 5(75) | 60 | F | NR | Austria | NR | NR | NR | NR | MG | NR | Phy | NR | Not checked | NR | NR | NR | NR | S |
| 143 | Stress from myasthenic crisis triggers Takotsubo (broken heart) syndrome case 6(75) | 64 | M | NR | Austria | NR | NR | NR | NR | MG | NR | Phy | NR | Not checked | NR | NR | NR | NR | D |
| 143 | Stress from myasthenic crisis triggers Takotsubo (broken heart) syndrome case 7(75) | 83 | F | NR | Austria | NR | NR | NR | NR | MG | NR | Phy | NR | Not checked | NR | NR | NR | NR | S |
| 103 | Stress-Induced Cardiomyopathy (Takotsubo Cardiomyopathy) After Liver Transplantation—Report of Two Cases(76) | 33 | F | NR | USA | AI,Other | Cardiopulmonary arrest | NR | AbN | PCS | NR | Phy | CKMB | E | T-invertion | N | 36 | 66 | S |
| 64 | Systemic lupus erythematosus cardiomyopathy - A case series demonstrating a reversible form of left ventricular dysfunction( case1)(77) | 49 | F | hispanic | USA | NR | NR | NR | NR | SLE | NR | Phy | NR | Not checked | NR | NR | 20 | 36 | S |
| 64 | Systemic lupus erythematosus cardiomyopathy - A case series demonstrating a reversible form of left ventricular dysfunction( case10)(77) | 40 | F | african american | USA | NR | Dyspnea | NR | NR | SLE | NR | Phy | NR | Not checked | NR | NR | 34 | 57 | S |
| 64 | Systemic lupus erythematosus cardiomyopathy - A case series demonstrating a reversible form of left ventricular dysfunction( case11)(77) | 31 | M | african american | USA | NR | NR | NR | NR | SLE | NR | Phy | NR | Not checked | NR | NR | 40 | NR | D |
| 64 | Systemic lupus erythematosus cardiomyopathy - A case series demonstrating a reversible form of left ventricular dysfunction( case12)(77) | 55 | F | caucasian | USA | NR | NR | NR | NR | SLE | NR | NR | NR | Not checked | NR | NR | 40 | NR | D |
| 64 | Systemic lupus erythematosus cardiomyopathy - A case series demonstrating a reversible form of left ventricular dysfunction( case13)(77) | 27 | F | caucasian | USA | NR | NR | NR | NR | SLE | NR | Phy | NR | Not checked | NR | NR | 30 | 60 | S |
| 64 | Systemic lupus erythematosus cardiomyopathy - A case series demonstrating a reversible form of left ventricular dysfunction( case14)(77) | 23 | F | african american | USA | NR | NR | NR | NR | SLE | NR | Phy | NR | Not checked | NR | NR | 42 | 55 | S |
| 64 | Systemic lupus erythematosus cardiomyopathy - A case series demonstrating a reversible form of left ventricular dysfunction( case2)(77) | 18 | F | caucasian | USA | NR | NR | NR | NR | SLE | NR | Phy | NR | Not checked | NR | NR | 30 | 54 | S |
| 64 | Systemic lupus erythematosus cardiomyopathy - A case series demonstrating a reversible form of left ventricular dysfunction( case3)(77) | 20 | F | hispanic | USA | NR | NR | NR | NR | SLE | NR | Phy | NR | Not checked | NR | NR | 45 | 55 | S |
| 64 | Systemic lupus erythematosus cardiomyopathy - A case series demonstrating a reversible form of left ventricular dysfunction( case4)(77) | 46 | F | african american | USA | NR | NR | NR | NR | SLE | NR | Phy | NR | Not checked | NR | NR | 24 | 64 | S |
| 64 | Systemic lupus erythematosus cardiomyopathy - A case series demonstrating a reversible form of left ventricular dysfunction( case5)(77) | 33 | F | caucasian | USA | NR | Dyspnea | NR | NR | SLE | NR | Phy | NR | Not checked | NR | NR | 46 | 61 | S |
| 64 | Systemic lupus erythematosus cardiomyopathy - A case series demonstrating a reversible form of left ventricular dysfunction( case6)(77) | 24 | F | african american | USA | NR | NR | NR | NR | SLE | NR | Phy | NR | Not checked | NR | NR | 30 | 45 | S |
| 64 | Systemic lupus erythematosus cardiomyopathy - A case series demonstrating a reversible form of left ventricular dysfunction( case7)(77) | 29 | F | african american | USA | NR | Dyspnea | NR | NR | SLE | NR | Phy | NR | Not checked | NR | NR | 19 | 50 | S |
| 64 | Systemic lupus erythematosus cardiomyopathy - A case series demonstrating a reversible form of left ventricular dysfunction( case8)(77) | 51 | F | caucasian | USA | NR | NR | NR | NR | SLE | NR | Phy | NR | Not checked | NR | NR | 25 | 60 | S |
| 64 | Systemic lupus erythematosus cardiomyopathy - A case series demonstrating a reversible form of left ventricular dysfunction( case9)(77) | 45 | F | african american | USA | NR | Dyspnea | NR | NR | SLE | NR | Phy | NR | Not checked | NR | NR | 31 | 55 | S |
| 80 | Tako Tsubo cardiomyopathy in a patient with antiphosholipid syndrome secondary to systemic lupus erythemathosus (SLE)(78) | 62 | F | NR | Malaysia | AI,Other | NR | NR | NR | SLE and antiphopholipid syndrome | NR | NR | NR | E | STE,Other,T-invertion | N | 35-40 | NR | S |
| 115 | Takotsubo (Ampulla-shaped) cardiomyopathy associated with microscopic polyangiitis(79) | 70 | F | NR | Japan | NR | NR | Fever | NR | Microscopic polyangitis (MPA) | P | Phy | ESR,CRP | E | T-invertion | NR | 24 | 78 | S |
| 81 | Takotsubo and Takayasu-A Reason to Rhyme?(80) | 51 | F | Chinese | Singapore | Other | Dyspnea,CP | NR | AbN | Takayasu arteritis | NR | NR | ESR | Not checked | STE | N | 50 | NR | S |
| 96 | Takotsubo Cardiomyopathy After Double-Lung Transplantation: Role of Early Extracorporeal Membrane Oxygenation Support(81) | 63 | F | AFRICAN AMERICAN | USA | CVD,AI,Other | Dyspnea | NR | NR | pulmonary sarcoidosis, connective tissue disease | NR | Phy | NR | E | N | N | 30 | N | D |
| 51 | Takotsubo cardiomyopathy and thrombotic thrombocytopenic purpura preceding a lupus diagnosis: A case report(82) | 60 | F | NR | Cyprus | NR | CP | NR | NR | SLE | P | NR | NR | E | STE,T-invertion | N | NR | 55 | S |
| 107 | Takotsubo Cardiomyopathy as a Manifestation of Dysautonomia in Guillain-Barré Syndrome: A Case Series and Review of the Literature case1(83) | 58 | M | NR | USA | Other | NR | Tachycardia | NR | axonal-variant GBS | NR | Phy | NR | Not checked | Other | NR | 15 | 65 | S |
| 107 | Takotsubo Cardiomyopathy as a Manifestation of Dysautonomia in Guillain-Barré Syndrome: A Case Series and Review of the Literature case 2(83) | 79 | F | NR | USA | Other | NR | NR | NR | AMSAN variant of GBS | NR | Phy | NR | Not checked | Other | NR | 30-35 | N | S |
| 82 | Takotsubo cardiomyopathy associated with autoimmune polyendocrine syndrome II(84) | 64 | M | NR | Japan | Other | CP | Hypotension, Fever | NR | autoimmune polyendocrine syndrome type II (APS II), Hashimoto’s disease | P | Phy | CKMB | Not checked | STE,Other,T-invertion | N | NR | NR | S |
| 133 | Pheochromocytoma-induced inverted Takotsubo cardiomyopathy: A case of patient resuscitation with extracorporeal life support(85) | 51 | F | NR | France | CVD,AI,Other | NR | Tachy cardia, high BP, | NR | crohn disease | NR | Phy | NR | E | STD | N | 30 | 72 | S |
| 87 | Takotsubo cardiomyopathy associated with Guillain-Barré syndrome: A differential diagnosis from dysautonomia not to be missed(86) | 60 | F | NR | France | CVD | NR | NR | NR | GBS | NR | Phy | NR | E | T-invertion | N | 45 | N | S |
| 19 | Takotsubo cardiomyopathy associated with levothyroxine over-replacement case 1(87) | 74 | F | NR | Portugal | AI | Dyspnea,CP | Hypotension | NR | auto-immune thyroiditis, rheumatoid arthritis | NR | Phy | NR | E | STE | NR | 43 | NR | S |
| 19 | Takotsubo cardiomyopathy associated with levothyroxine over-replacement case 2(87) | 47 | F | NR | Portugal | CVD,AI,Other | CP,Palplitation | NR | NR | Autoimmune thyroiditis | NR | Psy | NR | E | Other,T-invertion | N | NR | N | S |
| 52 | Takotsubo cardiomyopathy associated with Miller-Fisher syndrome(88) | 51 | F | NR | USA | NR | Dyspnea | Tachycardia, Tachypnea, Hypotension | NR | Miller-Fisher syndrome(GBS) | NR | Phy | NR | E | Other | NR | 35 | 55 | S |
| 148 | Takotsubo Cardiomyopathy Associated with Thyrotoxicosis: A Case Report and Review of the Literature(89) | 71 | F | NR | USA | Other | Syncope | Tachycardia, Tachypnea, Hypertension | NR | Graves disease | P | Phy | CKMB | E | Other | N | 30 | 47.5 | S |
| 76 | Takotsubo cardiomyopathy case series: Typical, atypical and recurrence(90) | 74 | F | Caucasian | UK | AI,Other | Dyspnea,CP | NR | NR | polymyalgia rheumatica and temporal arthritis | NR | Psy | NR | E | STE.Other | N | 41 | NR | S |
| 77 | Takotsubo cardiomyopathy complicated by Dressler's syndrome(91) | 75 | F | NR | Australia | AI,Other | CP | Tachycardia | NR | graves dx and Dressler’s syndrome | NR | Psy.Phy | NR | E | STE | N | NR | 72 | S |
| 104 | Takotsubo cardiomyopathy complicated with left ventricular thrombus in myasthenic crisis: A case report(92) | 67 | F | NR | Thailand | CVD,AI,Other | NR | NR | NR | MG | NR | Phy | NR | E | Other,T-invertion | N | 36 | 70 | S |
| 57 | Takotsubo cardiomyopathy complicating thyroidectomy for Graves' disease(93) | 40 | F | NR | Australia | AI | Dyspnea,CP | Tachycardia, Tachypnea | NR | Graves’ disease | P | Phy | NR | E | Other/T-invertion | N | NR | NR | S |
| 91 | Tako-Tsubo cardiomyopathy following colonoscopy: Insights on pathogenesis(94) | 60 | F | NR | USA | CVD,AI,Other | Syncope | Fever, Hypotension, Tachycardia | NR | GRAVES | NR | Phy | NR | E | STE | N | 15-20 | 75 | S |
| 108 | Takotsubo cardiomyopathy in a patient with Addison disease(95) | 71 | F | NR | USA | AI | CP,Cyanosis | Hypotension | NR | Addison | NR | ? | NR | E | STE |  | 25-30 | N | S |
| 22 | Takotsubo cardiomyopathy in a patient with Addison disease: Is apical ballooning always reversible?(96) | 40 | F | NR | Turkey | CVD,AI,Other | CP | Hypertension | AbN | Addisons disease | NR | NR | CKMB | E | Other | N | 44 | NR | S |
| 127 | Takotsubo cardiomyopathy in a patient with multiple autoimmune disorders and hyperthyroidism(97) | 75 | F | NR | Iran | CVD,AI,Other | Dyspnea,CP | NR | NR | Hashimoto thyroiditis with hyperthyroidism,pernicious anemia, and rheumatoid arthritis | NR | Phy | BNP,CKMB | E | NR | N | 30 | 55 | NR |
| 100 | Takotsubo cardiomyopathy in elderly F trauma patients: a case series(98) | 81 | F | Caucasian | UK | AI,Other | Syncope | Tachycardia, E BP | NR | MG | NR | Phy | BNP | E | STE,T-invertion | N | 25 | 64 | S |
| 59 | Takotsubo cardiomyopathy in myasthaenia gravis crisis confirmed by cardiac MRI(99) | 70 | M | NR | UK | AI,Other | Dyspnea,CP | NR | NR | myasthaenia gravis | P | Phy | NR | E | STE | N | NR | N | S |
| 70 | Takotsubo Cardiomyopathy in Myasthenic Crisis,case1(100) | 84 | F | NR | Australia | NR | Dyspnea | NR | NR | myasthenia gravis | P | Phy | NR | E | STE.T-invertion | N | NR | N | S |
| 70 | Takotsubo Cardiomyopathy in Myasthenic Crisis,case2(100) | 81 | M | NR | Australia | NR | Dyspnea | NR | NR | myasthenia gravis | P | Phy | NR | E | STE.T-invertion | N | NR | N | S |
| 70 | Takotsubo Cardiomyopathy in Myasthenic Crisis,case3(100) | 75 | M | NR | Australia | NR | Dyspnea | NR | NR | myasthenia gravis | P | Phy | NR | E | STE.T-invertion | N | NR | N | S |
| 70 | Takotsubo Cardiomyopathy in Myasthenic Crisis,case4(100) | 74 | F | NR | Australia | NR | Dyspnea | NR | NR | myasthenia gravis | P | Phy | NR | E | STE.T-invertion | N | NR | N | S |
| 111 | Takotsubo cardiomyopathy in the setting of a myasthenic crisis(101) | 77 | M | NR | USA | CVD,Other | Dyspnea | Hypotension | NR | Myasthenia gravis | NR | Phy | NR | E | N | N | 25-30 | 60 | S |
| 20 | Takotsubo cardiomyopathy in the setting of necrotizing myopathy (102) | 61 | F | NR | canada | AI,Other | Dyspnea,CP,Syncope | Fever | NR | necrotizing myopathyNecrotizinig myopathy | P | NR | ESR,CRP | E | STE,Other | N | 30–35 | 63 | S |
| 37 | Takotsubo cardiomyopathy related to compound electrolyte aberration and anemia in Crohn's disease(103) | 61 | F | NR | Poland | AI,Other | CP | Fever | NR | Crohn's disease,inflammatory intestine tumor(?) | NR | Phy | CKMB | E | STE,Other,T-invertion | N | 35 | 50 | S |
| 53 | Takotsubo Cardiomyopathy with Guillain-Barré Syndrome(104) | 70 | F | NR | USA | Other | CP | NR | NR | Guillain-Barré syndrome | NR | Phy | NR | E | N | N | 30 | 50 | S |
| 128 | Takotsubo Cardiomyopathy: Is It Due to Adrenal Crisis or Sjogren's Flare?(105) | 64 | F | Caucasian | USA | CVD,AI | NR | Hypotension | NR | Sjogren’s disease a | NR | Phy | BNP,CKMB | E | T-invertion | N | 20-25 | 50 | S |
| 114 | Takotsubo Myocardiopathy and Hyperthyroidism: A Case Report and Literature Review(106) | 34 | M | NR | Argentina | CVD | Dyspnea,CP | Tachycardia | NR | Graves’ disease | P | Psy,Phy | NR | E | T-invertion | N | 40 | NR | S |
| 73 | Takotsubo Syndrome After Cardiac Surgery(107) | 62 | F | NR | Israel | CVD,AI | Dyspnea | Tachycardia, Hypotension | NR | rheumatic heart disease | NR | Phy | NR | E | Other.T-invertion | N | 35 | 55 | S |
| 84 | Takotsubo syndrome after heart valve surgery(108) | 68 | F | NR | Spain | CVD,AI,Other | Dyspnea,Palplitation | Tachycardia, Tachypnea | AbN | rheumatic heart disease | NR | Phy | NR | E | Other.T-invertion | N | 35 | NR | S |
| 69 | Takotsubo syndrome associated with autoimmune limbic encephalitis: a case report(109) | 68 | F | Japanese | Japan | Other | Decreasd LOC | Tachycardia | NR | autoimmune hypothyroidism, autoimmune limbic encephalitis | Neg | Phy | BNP,CKMB | E | STE | N | 28 | 69 | S |
| 42 | Takotsubo syndrome associated with myasthenic crisis. A case report(110) | 69 | F | NR | Mexico | CVD,AI | Dyspnea,CP | Tachycardia, Tachypnea | AbN | myasthenia gravis | NR | Phy | CKMB | E | STE,T-invertion | N | NR | 76 | S |
| 125 | Takotsubo Syndrome during Haemodialysis(111) | 55 | F | NR | Greece | AI,Other | CP | Hypotension | NR | vasculitis and inflammatory bowel disease | NR | Phy | CKMB | N | STE,T-invertion,Other | N | 15 | N | S |
| 49 | Takotsubo Syndrome in a Rheumatoid Arthritis Patient Under Tofacitinib: A Case Report(112) | 57 | F | white | Portugal | AI,Other | CP | NR | NR | RA | P | Phy | CKMB | E | Other/T-invertion/STENRSTESTE | N | 38 | 55 | S |
| 86 | Takotsubo syndrome: hyperthyroidism, pheochromocytoma, or both? A case report(113) | 69 | M | NR | Italy | CVD,AI,Other | Dyspnea,CP | Hypertension/Tachycardia | NR | Graves’s disease | P | Phy | NR | E | Other,T-invertion | N | 23 | 50 | S |
| 88 | Tako-tsubo-like syndrome in systemic sclerosis: a sign of myocardial Raynaud phenomenon?(114) | 84 | F | NR | Italy | AI,Other | NR | Fever | NR | limited cutaneous systemic sclerosis (SSc) | NR | NR | NR | E | T-invertion | N | 35 | 65 | S |
| 34 | Takotsubo-like syndrome triggered by fludrocortisone overdose for Addison's disease: a case report(115) | 41 | F | White | Austria | AI,Other | Syncope | Tachycardia, Tachypnea | AbN | ,autoimmune Hashimoto thyroiditis, Adrenal insufficiency ( the diagnosis of PGA II was suspected) | NR | Phy | NR | E | Other,T-invertion | NR | 30 | N | S |
| 38 | The brain-heart connection: A multiple sclerosis relapse presenting as Takotsubo Syndrome. A case report and literature review(116) | 42 | F | NR | Italy | NR | NR | NR | NR | MS | NR | Phy | CKMB | E | STD,Other | N | 40 | N | S |
| 13 | The nervous heart: A case report and discussion of an under-recognized clinical problem(117) | 73 | F | Cacausian | UK | NR | Dyspnea | NR | AbN | Guillain–Barre´ syndrome | NR | Phy | NR | E | STD,Other,T-invertion | N | NR | N | S |
| 132 | Transient left ventricular apical ballooning syndrome following a hyponatraemic seizure(118) | 69 | F | NR | Canada | AI,Other | NR | NR | NR | MS | NR | Psy,Phy | NR | ??? | STE | NR | NR | N | NR |
| 30 | Transoesophageal echocardiography: An unusual trigger to Takotsubo cardiomyopathy(119) | 65 | F | NR | France | CVD,AI | Dizziness | NR | NR | rheumatic heart disease | NR | Phy | NR | E | STE | NR | 20 | NR | S |
| 126 | Typical and reverse Takotsubo syndromes as initial manifestations of consecutive Addisonian crises in a 38-year-old patient: The heart has its reasons!(120) | 38 | F | NR | Canada | AI | Syncope | Hypotension, Tachycardia | AbN | Auto-Immune Polyglandular Syndrome Type II (APS II)/adernal crisis | NR | Phy | BNP  ,CKMB | E | Other | NR | 10–15 | 70 | S |
| 118 | Ventriculoseptal Rupture Caused by Takotsubo Syndrome(121) | 80 | F | NR | USA | CVD,AI,Other | Dyspnea,CP.pre syncope | NR | NR | RA | NR | NR | NR | E | STE,Other,T-invertion | N | 40-45 | 60 | S |

AI: autoimmune, NR: not reported, F: Female, M: Male, N: Normal, E: Elevated, S: Survived, D: Died, CP: Chest Pain, Psy: Psychological, Ph: Physical, Neg: Negative, P: Positive, CC: chief complaint, CVD: cardiovascular disease, STD: ST depression, STE: ST elevation
